# Primary caregiver Reports of Patient-Initiated and Suffered Violence in Schizophrenia: A cross-sectional study in Tunisia

**DOI:** 10.1192/j.eurpsy.2023.964

**Published:** 2023-07-19

**Authors:** M. Stambouli, F. Fekih Romdhane, F. Ghrissi, W. Cherif, M. Cheour

**Affiliations:** Psychiatry department E, Razi hospital, Manouba, Tunisia

## Abstract

**Introduction:**

Aggressive behavior in psychosis is not uncommon. Studies found that among the patients with schizophrenia engaged in violence, more than half committed violence directly against family members. However, few studies have explored violence victimization and perpetration among caregivers of patients with schizophrenia.

**Objectives:**

Our study investigated caregiver reports of aggressive acts committed or suffered by their relative with schizophrenia

**Methods:**

This is a cross-sectional study among caregivers of patients with schizophrenia during the period from June to August 2022.Patients who attended our department of psychiatry at the Razi.The questionnaire was divided into three sections.

The first section: contained items regarding patient and caregiver-related information.

The second section: caregivers were asked questions about their experience of violence perpetration and victimization involving their relative with schizophrenia in the past 12 months.Beyond frequency, caregivers were also asked to specify, the causes of the violence perpetrated and suffered.

The third section contained two measures: The Depression Anxiety and Stress Scales (DASS-21) and the abridged version of the Zarit Burden Interview (ZBI), assessing psychological distress and caregiving burden, respectively.

**Results:**

Finally 110 caregivers were included ,the majority of caregivers were females (63.6%) and consisted of patients’ parents (50.9%). Verbal violence was the most reported type of violence victimization (35.5%). In addition, 54.5% of caregivers disclosed having perpetrated verbal violence at least once against their ill relative.

Bivariate analysis showed that lower caregivers’ educational level (p=.017), unemployment (p<.001), other person in charge (p=.027), burden levels (p<.001), depression (p<.001), anxiety (p<.001) and stress (p<.001) symptoms are positively associated with violence victimization occurrence. while being male caregiver (p=.007), having other person in charge (p<.001) and higher levels of depression (p<.001), anxiety (p<.001), and stress (p<.001) were associated with more violence perpetration.

**Conclusions:**

Our findings suggested that violence victimization and perpetration in schizophrenia are not uncommon.Appropriate procedures for minimizing it should be considered.

**Disclosure of Interest:**

None Declared

